# Cases of Diseased Testicle

**Published:** 1826-11

**Authors:** 

**Affiliations:** St. George's Hospital.


DISEASES OF THE TESTICLE.*

*Cases of Diseased Testicle.* Treated by Mr. Jeffreys, at St. George's Hospital.

Case I. *of Hydrocele, where a simple puncture with a lancet was followed by suppuration within the Tunica Vaginalis, and a radical cure of the disease.*

James Whitton, ætatis forty-five, was admitted into St. George's Hospital, April 6th, 1825, with a tumor of considerable size in the left side of the scrotum. He said that he had had a swelling on

* The continuation of Mr. Brodie's paper on this subject will be given in our next. (Editor.)

that part for four years, but had suffered little or no inconvenience from it until about four or five days ago, when, without any evident cause, it suddenly increased in size, and became very tense and painful, and of a darker colour than usual. He added, that he had formerly been the subject of hydrocele in the right tunica vaginalis, which was cured by injection two years before the present tumor appeared.

The tumor was now nearly of the size and shape of a small ostrich's egg, tense, painful, and slightly discoloured, as if from the effusion of blood within the tunica vaginalis. The pain extended to the groin and back; and the cord was fuller than natural. A fluctuation could be felt in it, but it was so obscure as to leave a doubt in the minds of some who examined it, whether the disease was hydrocele or pulpy testicle. The man, however, appeared to be in full health, and the history of the case was more like that of hydrocele.

He was ordered to keep his bed, to take some house-physic, and apply cold spirit-lotion to the scrotum.

April 8th.—A puncture was made with a lancet in the most depending part of the tumor, and about ten ounces of serous fluid, a good deal tinged with blood, but without any coagula, were evacuated. The testicle was found to be in a healthy condition, and of its natural size. The man remained in bed on low diet, and continued the use of the cold lotion, with a bag-truss.

In a few days the fluid accumulated again in the ordinary manner, and the scrotum acquired nearly its former size; but nothing occurred out of the usual course of events until the 16th. On that day he was seized with an attack of inflammation in the tunica vaginalis and testicle, accompanied with increase of swelling, and a considerable degree of pain in the part.

Ten leeches were applied to the tumor, and the bleeding was encouraged by the poppy fomentation.—In the evening, the same number of leeches was again put on, and he took some Dover's powder.

On the following day (the 17th), the scrotum was still very much swollen and inflamed, and he complained of severe pain.

Ten ounces of blood were drawn from his arm; and he was ordered to take a saline draught, containing a drachm of Sulphate of Magnesia and half a drachm of Vinum Antimonii Tartarizati, every four hours.—A linseed poultice was applied over the scrotum.

On the 20th, the puncture which had been made with the lancet opened afresh, and gave vent to a quantity of bloody serum, by which the pain and inflammation were much relieved.

25th.—A glairy fluid, mixed with pus, continued to ooze from the puncture. A probe, introduced at the opening, could be passed nearly its whole length into the cavity of the tunica vaginalis. The swelling of the scrotum had a good deal diminished.

May 2d.—Pure pus only was now discharged from the puncture, and there was no collection of fluid within the tunica vaginalis. The testicle remained in an indolent state: it was about the size of a duck's egg, smooth and uniform in its figure, somewhat hard, but free from pain.

A drachm of the Unguentum Hydrargyri cum Camphorâ was directed to be rubbed over the scrotum once a-day, before putting on the poultice.

Under this treatment, the swelling and induration of the testicle gradually subsided. On the 20th, the puncture in the scrotum was healed; and, on the 1st June, he was discharged cured. The cavity of the tunica vaginalis appeared to have been obliterated, by the cohesion of that membrane with the tunica albuginea.

Case II. *of Extirpation of a Testicle, in which an unusual combination of morbid structure was observed.*

James Gibbons, an agricultural labourer, twenty-seven years of age, was admitted February 1st, 1826, on account of a swelling of considerable magnitude in the left side of the scrotum, occasioned by a disease of the testicle. The tumor was of an oblong, oval shape, resembling a cocoa-nut, and about six inches in length by four inches in breadth. It was perfectly smooth and uniform on its surface, and had a firm elastic feel when examined, which in some parts resembled that arising from an indistinct fluctuation of fluid. The epidydimis could not be distinguished from the body of the testicle. The cord was slightly enlarged, but neither indurated nor painful. The integuments of the scrotum had a natural appearance, except that the cutaneous veins were larger and more numerous than usual, and they were loose and moveable over the surface of the tumor. There was a pricking shooting pain through the testicle, which at first, he said, used to occur only occasionally, and sometimes not for a month together; but during the last three months it had become more constant and severe, and now extended to his loins. He had of late been losing flesh, had a sallow complexion, and looked out of health.

He stated, that he had first observed a swelling in that side of his scrotum, between two and three years before; that it acquired nearly its present size in the course of a few days; that it was not at first attended with much pain; and that he had been able to follow his usual occupations until within the last three months.

He was kept in bed on low diet; and, at the end of a week, in order to remove any doubts as to the nature of its contents, a puncture was made in the lower part of the tumor, but only a small quantity of pale, pulpy matter, resembling soft brain, and a little blood, escaped. The puncture healed immediately.

Feb. 13th.—The testicle was extirpated. A single incision was made, beginning an inch above the ring of the external oblique muscle, and terminating at the bottom of the tumor; the cord was secured with two ligatures, in the manner recommended by Sir E. Home, in his work on Cancer; and three or four small arteries were tied in the scrotum. The edges of the wound were then brought together with two sutures, and covered with lint, over which were placed compresses of linen, wetted with spirit-lotion, and the whole was secured with a T bandage.

The diseased mass, immediately after its removal, weighed exactly one pound avoirdupois. On being laid open by an incision carried through the middle of the tumor, about a drachm of yellow serum escaped from under the upper part of the tunica vaginalis. This was the only part at which that membrane did not firmly adhere to the testicle. In the centre of the divided mass was a dark, purple-coloured, fungoid structure, about the size of a walnut, resembling fungus hæmatodes: around this was a half-fluid substance, in appearance like softened brain or marrow; and beyond this the tumor had very much the character of schirrus, being hard and fibrous, and it was interspersed with numerous bony spiculæ; and a number of small cysts or hydatids, varying in size from a pin's head to that of a dried bean, and containing a yellow water. At the upper, and also at the lower part of the tumor, there still remained portions of what appeared to be the original structure of the testicle, completely separated from each other by the intervening mass of disease.

17th. —The sutures in the wound were cut out, and the ligatures came away from the arteries in the scrotum. Wound healthy.

24th, (twelfth day from the operation.)—The ligatures from the cord came away. Wound healing kindly.

March 14th.—The wound all but healed.

22d.—Discharged cured.

---

